# Oxygen supplementation and cognitive function in long-COVID

**DOI:** 10.1371/journal.pone.0312735

**Published:** 2024-11-05

**Authors:** Christine Gagnon, Thomas Vincent, Louis Bherer, Mathieu Gayda, Simon-Olivier Cloutier, Anna Nozza, Marie-Claude Guertin, Patricia Blaise, Isabelle Cloutier, Alan Kamada, Stanislav Glezer, André Denault, Jean-Claude Tardif

**Affiliations:** 1 Montreal Heart Institute, Université de Montréal, Montreal, Quebec, Canada; 2 Montreal Health Innovations Coordinating Center (MHICC), Montreal, Quebec, Canada; 3 Inogen, Goleta, California, United States of America; Purdue University, UNITED STATES OF AMERICA

## Abstract

**Background:**

Patients can experience persistent cognitive complaints and deficits in long-COVID. Inflammation and capillary damage may contribute to symptoms by interfering with tissue oxygenation.

**Methods:**

This was an exploratory pilot crossover study designed to describe the effects of supplemental oxygen (portable oxygen concentrator, POC) on cognitive performance and peripheral and cerebral oxygen saturation at rest and exercise. Participants with long-COVID (n = 21) were randomized 1:1 to: 1) POC (3h/day) for 2 weeks followed by standard of care (Control) for 2 weeks or 2) Control for 2 weeks then POC (3h/day) for 2 weeks, with a 1-week washout. Cognitive assessment (global cognition [Montreal Cognitive Assessment, MoCA], episodic memory [Hopkins], working memory [Digit Span], executive function [Verbal fluency]) was performed at baseline and after each treatment period. Patient Health Questionnaire (PHQ-9) and Generalized Anxiety Disorder-7 were completed. Peripheral and cerebral oxygen saturation were measured at rest and exercise (treadmill) at baseline and after each treatment period. Statistical analyses were descriptive without formal testing.

**Results:**

MoCA scores were similar under POC (26.45±2.31) and Control (26.37±2.85); overall POC-Control difference was -0.090 (95% CI [-1.031, 0.850]). Because of a learning effect, post-hoc analyses were performed for Period 1, where the MoCA score difference was 1.705 [0.140, 3.271]. MoCA subscores suggested better performance with POC for Visuospatial/executive (0.618 [-0.106, 1.342]) and Attention (0.975 [0.207, 1.743]). POC trended to have better scores on Digit Span backward (difference: 0.822 [-0.067, 1.711]) and self-reported depressive symptoms (difference: -1.335 [-3.166, 0.495]). For specific PHQ-9 items, POC tended to have lower (better) scores for Q1 (Little interest/pleasure) and Q7 (Trouble concentrating). Cerebral oxygen saturations at end of exercise showed no difference between POC and Control. Peripheral saturations during exercise were similar under POC and Control (difference: 0.519% [-1.675, 2.714]).

**Conclusion:**

An advantage of POC over Control was observed for global cognition, attention, visuospatial/executive performance and depressive symptoms. Results need to be validated in a larger study.

## Introduction

It is estimated that at least 10% of individuals that have been infected by SARS-CoV-2 will develop long-COVID [1], defined as symptoms that occur 3 months from the onset of SARS-CoV-2 infection, that last for at least 2 months, and that cannot be explained by another diagnosis according to the World Health Organization [2]. Long-COVID is heterogeneous and encompasses a broad range of manifestations; it can affect multiple organ systems, including the respiratory, cardiovascular and neurological systems. Cognitive impairment is a major feature of long-COVID with deficits in attention, executive functions and memory, as well as “brain fog” as the most consistently reported in the literature [3]. Neuropsychiatric symptoms such as depression and anxiety are also common in long-COVID. The mechanisms underlying cognitive impairment in long-COVID are still being investigated, yet many hypotheses have been proposed, including direct viral invasion of the central nervous system, neuroinflammation, vascular dysfunction, and the indirect effects of systemic inflammation and immune dysregulation. Also, impaired oxygenation resulting from SARS-CoV-2-related inflammation and capillary damage may play a significant role in the development of both cognitive dysfunction and depression in individuals with long-COVID [4]. Finally, factors such as psychological distress, sleep disturbances, and burden of illness may contribute to cognitive impairment in long-COVID [5]. Although there are many ongoing studies on interventions for long-COVID management, there is to date no validated treatment. Oxygen therapy (hyperbaric chamber) showed promising pilot results by improving long-COVID symptoms (fatigue) and cognition in adults [6]. The accessibility of this approach can be limited, but portable oxygen supplementation may be a potential alternative therapy in long-COVID management that has not yet been studied. The potential benefit of oxygen therapy on cognition is thought to be by improving chronic hypoxemia observed in long-COVID patients [6].

The primary objective of this pilot exploratory study was to describe the effects of portable oxygen therapy on cognitive performance using the Montreal Cognitive Assessment (MoCA) and a battery of neuropsychological tests, as well as on cerebral and peripheral oxygen saturation. Secondary objectives included the description of the effects of portable oxygen therapy on post-COVID functional status and psychological health.

## Methods

### Trial design

**Fig 1:**
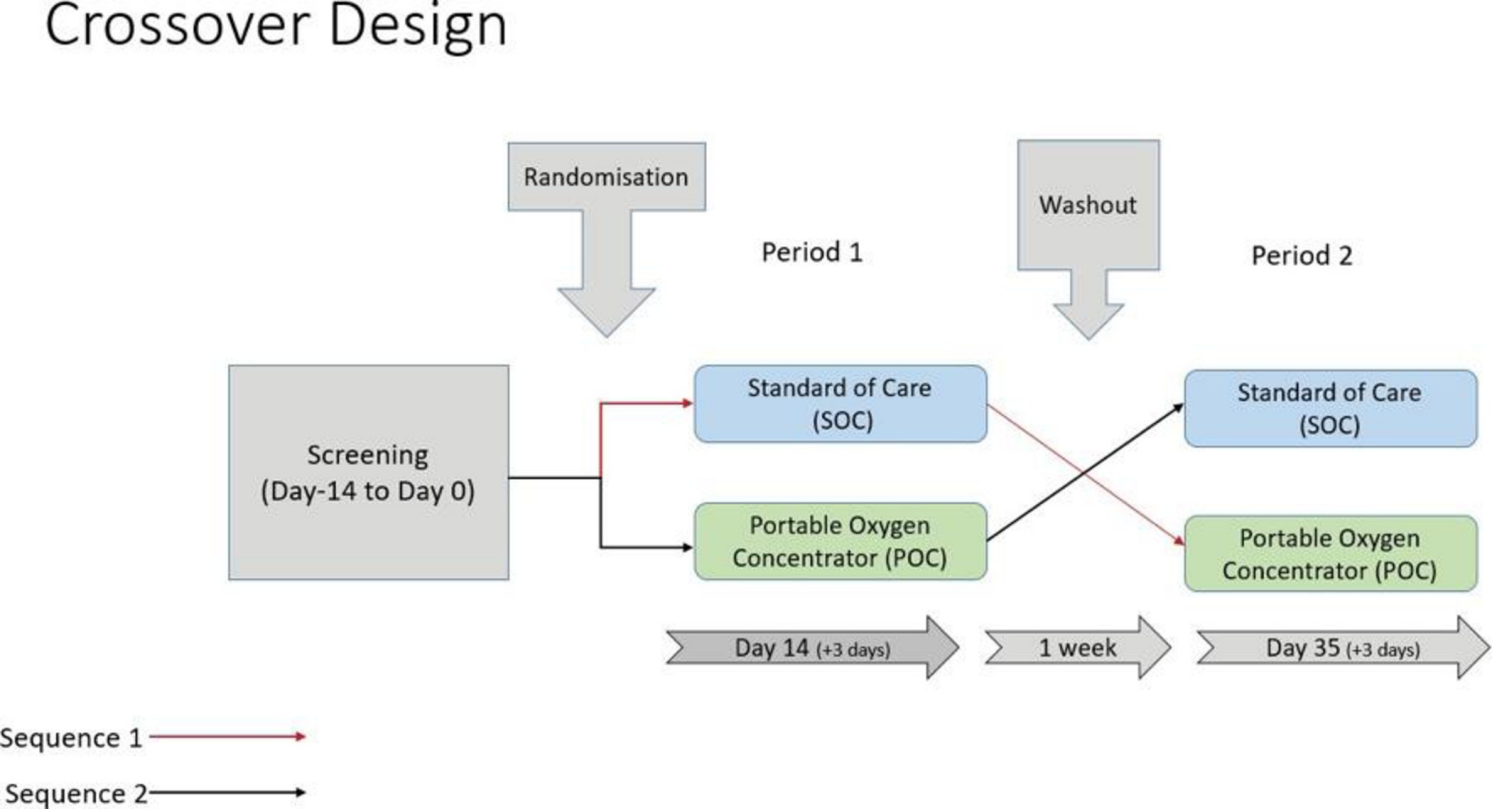
Study design.

This was a pilot, exploratory, crossover randomized study. Participants underwent a 2-week screening/baseline assessment following which they were randomly allocated to one of the following arms (1:1 allocation ratio): 1) Sequence 1 (POC-Control): portable oxygen concentrator (POC) for a first 2-week period followed by a 2-week standard-of-care (Control) period or 2) Sequence 2 (Control-POC): Control for the first 2-week period, followed by POC for the second 2-week period. A 1-week washout period was held between the 2-week treatment periods (Fig 1).

The Inogen One® G4 POC supplied a high concentration of oxygen and was used with a nasal cannula that channeled oxygen from the concentrator to the patient. Participants were instructed to start flow setting at 3, which could be reduced based on investigator judgment. Participants had to use the POC for a minimum of 3 hours per day. Standard of care (Control) was defined as the participant living as usual, with their symptoms or complications-based treatment and without any further intervention.

Patients managed for their clinical care were referred from the Montreal Heart Institute (Montreal, Canada) and from long-COVID clinics in the Montreal area. The Montreal Heart Institute’s research ethics board approved the project (protocol number 2022–3083) on March 13^th^ 2022. The study was conducted in accordance with the Helsinki Declaration and in accordance with Good Clinical Practice guidelines. All investigators and research personnel were trained and certified in good clinical practices and ethical courses. The recruitment period was initiated on June 8^th^ 2022 and completed on December 14^th^ 2022. The language used by patients was French.

### Study population

Adult patients were eligible if they were diagnosed with long-COVID, had self-reported concerns regarding cognitive functioning (using the Subjective Cognitive Impairment Questionnaire) or a recent diagnosis of COVID-19 related cognitive impairment, were able to perform an exercise treadmill test at screening, tolerated pulsed oxygen therapy delivered by POC at screening, and were willing and able to use the POC system. Patients were excluded if they had a contraindication to the use of POC including allergy to cannula material, were pregnant or planning to become pregnant during the study, had non-COVID-related cause for cognitive impairment, had a respiratory infection at screening or randomization, or participated in another interventional clinical trial within 30 days of randomization.

### Study procedures

Prior to engaging in the study, all participants had provided written informed consent. The screening and baseline evaluations were performed on two separate visits. The Subjective Cognitive Impairment Questionnaire was completed during the first visit [7]. Trained doctoral students in neuropsychology (supervised by certified and experienced neuropsychologists) then administered the Montreal Cognitive Assessment (MoCA), other neuropsychological tests, as well as psychological health and long-COVID questionnaires as described below. During the second baseline visit, participants completed a cardiopulmonary exercise treadmill test, blood tests, and post-COVID-19 functional scale (PCFS) evaluation; near-infrared spectroscopy (NIRS) brain oximetry and fingertip pulse oximetry were performed to measure local and systemic oxygen saturation at rest and during treadmill exercise. The assessments described in the previous paragraph were repeated at the end of both study periods (POC-Control or Control-POC).

### Endpoints and measures

The following tests and questionnaires were performed at baseline and following each 2-week treatment period, unless specified otherwise. Participants used their POC during the treadmill exercise test and the MoCA only when testing occurred after their POC period.

#### Cognitive tests and questionnaires

Montreal Cognitive Assessment (MoCA). The MoCA is a cognitive screening test that is extensively used in clinical and research settings [8, 9]. Its subscores target different cognitive domains: visuospatial/executive functions, naming, attention, abstraction, memory and orientation. In order to mitigate the potential issue of a learning effect over a relatively short period of time, version 1 of the MoCA was used at the baseline and end-of study visits, whereas version 2 was used at the end of the first 2-week treatment period. Both versions of the MOCA have previously been validated. The total MoCA score on 30 was analyzed, as well as its subscores (visuospatial/executive /5; naming /3; attention /6; language /3; abstraction /2; delayed recall /5; orientation /6).

Hopkins Verbal Learning Test (HVLT). During the HVLT, participants were read a 12-word list. They were instructed to listen carefully and to recall as many words as possible, in any order. During the learning phase, the wordlist was read 3 times in total. After a 20-minute delay, and without being warned beforehand, participants were asked to recall as many words as possible. The variables of interest for the HVLT were the total of words recalled during the learning phase (total words; /36) and the total words recalled after the 20-minute delay (delayed recall; /12) [10].

Digit Span. The Digit Span is a subtest of the Weschler Adult Intelligence Scale (WAIS-IV) [11]. During the Digit Span Forward, participants were instructed to repeat in the same order digit series that were presented to them orally. This assesses verbal short-term memory. During the Digit Span Backward, participants were read digit series that they had to repeat in reverse order. This part of the test assesses working memory. Variables of interest for this test were the total points for Digit Span Forward (/16) and the total points for Digit Span Backward (/16).

Verbal Fluency. During this test, participants were asked in the first place to name as many words as possible that start with the letter P (phonological fluency). Second, they were asked to name as many animals as possible in 60 seconds.

Subjective Cognitive Impairment Questionnaire. In this questionnaire, participants had to answer 4 questions related to subjective cognitive impairment. The questionnaire was based on a publication by Jessen and collaborators (2020). Q1: *Do you find that your memory is poorer than it was 5 years ago*? (No, Yes but it doesn’t worry me, Yes and it worries me). Q2: *Do you find that your thinking abilities are poorer than they were 5 years ago*? (No, Yes but it doesn’t worry me, Yes and it worries me); Q3: *Have you consulted a physician for the changes in your memory and thinking*? (yes/no); Q4: *Do you think that people who know you well would say that your memory is* (the same as it was 5 years ago/ slightly poorer than it was 5 years ago/ moderately poorer than 5 years ago/ significantly poorer than 5 years ago?).

General Anxiety Disorder-7 (GAD-7). The GAD-7 enquires on the presence and severity of anxiety symptoms in the previous 2 weeks [12]. The variable of interest of this questionnaire was the total score (/21).

Patient Health Questionnaire-9 (PHQ-9). The PHQ-9 assesses the presence and severity of symptoms of depression in the previous 2 weeks (/27) [13]. Questions 1 and 7 (“little interest/pleasure” and “trouble concentrating”) were also analyzed individually, as they were hypothesized to be more sensitive to long-COVID.

Post-COVID Functional Scale (PCFS). This questionnaire assesses the extent to which long-COVID symptoms impact everyday life on different functional outcomes. Grade 0/ no limitations; Grade 1/ negligible limitations in everyday life; Grade 2/ occasional limitations in everyday life; Grade 3/ limitations in everyday life, unable to perform all activities but does not require assistance for self-care; Grade 4/ Severe limitations in everyday life, needs assistance and care [14].

Long-COVID-19 symptoms questionnaire. This questionnaire was completed only at baseline and interrogated participants on the presence and severity of various long-COVID symptoms. The questionnaire was based on the WHO’s Global COVID-19 Clinical Platform: Post-COVID-19 condition CRF [15].

#### Exercise stress tests on treadmill

Participants were first asked to rest on a chair for 3 minutes and then stand up to undergo a maximal exercise test on a treadmill (Trackmaster, TMX428, USA) with individualized Ramp protocols where speed and slope progressively increased to achieve a linear load and an exercise duration of approximately 10 minutes. There was continuous ECG monitoring (Norav, Stress1000W, Israel) and blood pressure was measured every 2 minutes with a semi-automated device (Tango M2, Suntech, China). The test was stopped if there was inability to maintain the walking speed, subject exhaustion with cessation caused by fatigue and/or other clinical symptoms (dyspnea, abnormal blood pressure responses) or ECG abnormalities that required exercise cessation [16]. During the baseline visit only, gas exchanges were measured continuously at rest, during exercise, and after exercise cessation using a metabolic system (Cosmed Quark b2, Italy, Rome). The calibration of the flow module was accomplished by introducing a calibrated volume of air at several flow rates with a 3-liter pump. Gas analyzers were calibrated before each test using a standard certified commercial gas preparation (O_2_: 16%; CO_2_: 5%) as previously published. Data were measured every four respiratory cycles during testing and then were averaged every 15 seconds for minute ventilation (VE, in L/min, BTPS), oxygen uptake (VO_2_, in L/min, STPD), and carbon dioxide production (VCO_2_, in L/min, STPD). The highest VO_2_ value reached during the exercise phase of each test was considered as the VO_2_ peak. Other key variables like VE/VCO_2_ slope and OUES were also calculated. Participants only wore the POC device during the exercise test that was performed after their POC 2-week period.

#### Cerebral and peripheral oxygen saturation

Cerebral Near Infra-Red Spectroscopy (NIRS). The NIRS device (O3 Regional Oximetry–company: Masimo; Irvine, California) tracked oxygen saturation (rSO2) as well as absolute changes for oxyhemoglobin (ΔO_2_Hb), deoxyhemoglobin (ΔHHb) and total hemoglobin (ΔcHb) [17]. Two sensors were symmetrically applied on the participant’s forehead, above the eyebrows. The main measures consisted of left and right rSO2 values at the end of the rest period and at the end of the exercise treadmill test. In an exploratory manner, the full NIRS time-series for rSO2, ΔO_2_Hb, ΔHHb and ΔcHb were recorded during the exercise test at a sample rate of 0.5 Hz. Non-distorting noise reduction was applied to the time-series using the Savitzky–Golay filter with a window length of 21 and an order of 2 [18]. To allow temporal alignment of time-series corresponding to varying effort durations, time-series were temporally normalized to fit an effort progression scale between 0 and 100%.

Peripheral oxygen saturation. This was measured via fingertip pulse oximetry at the end of the rest period and at the end of the exercise test.

### Statistical analyses

The statistical analyses were planned to be descriptive and to provide estimates of the treatment effect with 95% confidence intervals. No formal statistical hypotheses were tested in this pilot exploratory study. A repeated analysis of variance (ANOVA) model was used to estimate the treatment effect. Specifically, the model accounted for treatment group (Control, POC), sequence of randomization (sequence 1, sequence 2; see Fig 1) and period (period 1, period 2). From this model, an estimate of the difference between Control and POC was obtained and presented with a 95% confidence interval. For Period 1 only, an analysis of covariance (ANCOVA) model was used to estimate the treatment effect. The model accounted for treatment group (Control, POC) and the baseline value. Differences between groups were estimated and presented with a 95% confidence interval. The post-COVID-19 functional status scale (PCFS) was assessed on a categorical scale and analyzed using a repeated proportional-odds cumulative logit model, accounting for treatment group, sequence of randomization and period. The odds ratio (OR) will be presented with a 95% confidence interval. All analyses were conducted in the intent-to-treat (ITT) population. Statistical analyses were performed using SAS Version 9.4. Analyses were performed by biostatisticians from the Montreal Health Innovations Coordinating Center (MHICC, Montreal, Canada).

### Results

**Fig 2:**
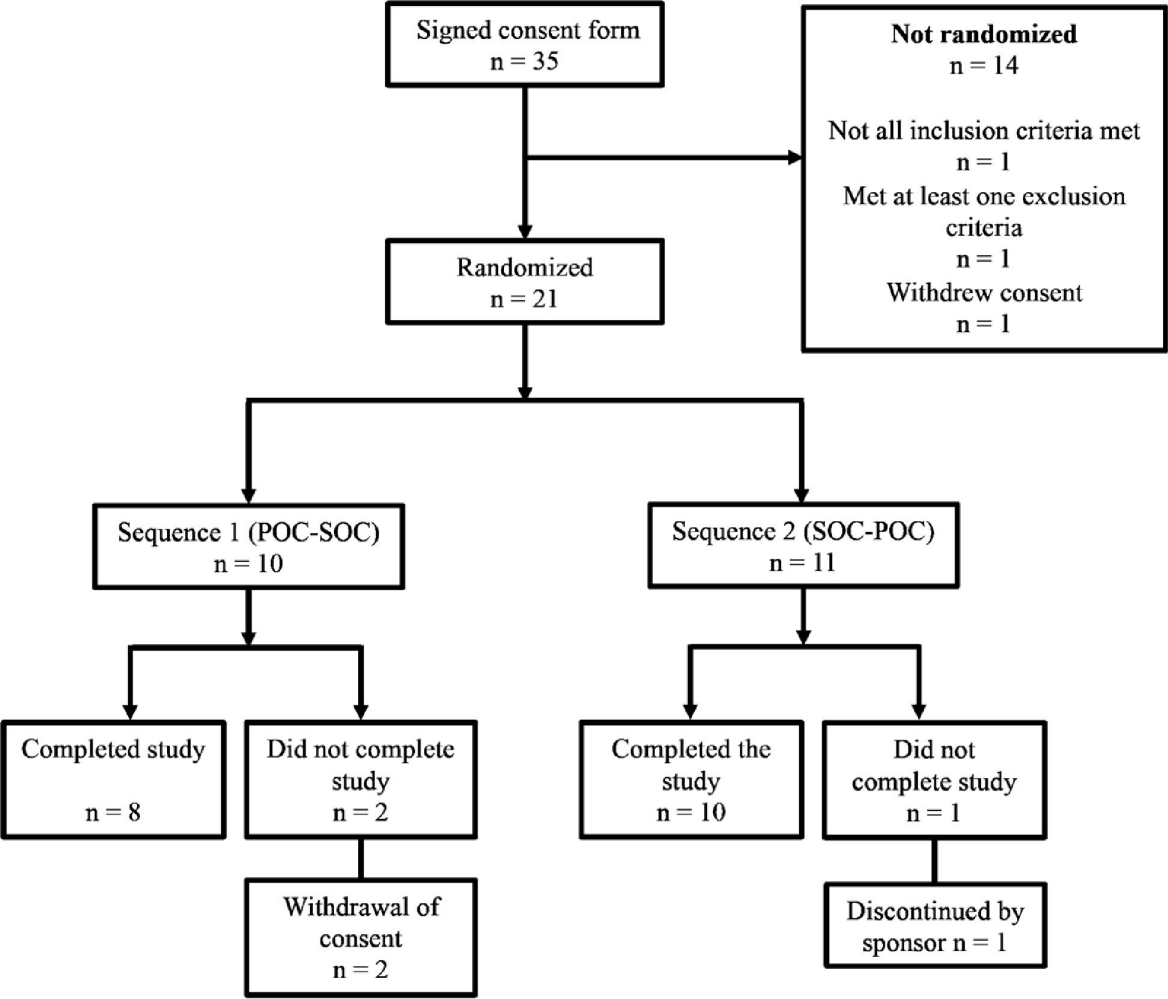
Flow diagram.

A total of 35 patients were recruited for this study, from which 21 (17 female and 4 male participants) were randomized (10 in Sequence 1 and 11 in Sequence 2). In Sequence 1 (POC-Control), 8 participants completed the study, whereas in Sequence 2 (Control-POC), 10 participants completed the study (the flow diagram is shown in Fig 2). The median age of randomized participants was 47.7 years (IQR: 40.6, 54.1). No serious adverse events occurred during this study. On the Long-COVID questionnaire, a majority of participants reported *still present* or *intermittently present* the following symptoms: discomfort after exertion (19/21), fatigue (21/21), shortness of breath (19/21), memory problems (21/21), and concentration disorders (21/21). On the Subjective Cognitive Impairment Questionnaire, 100% of participants reported worries with their memory and thinking abilities (21/21) and 81% reported having consulted their physician for this reason (17/21). The participants’ demographic characteristics are shown in Table 1.

**Table 1:** Demographic characteristics of study participants.

Characteristics	All (N = 21)
	Median (Q1, Q3)/ Count (percentage)
**Age (years)**	47.7 (40.6, 54.1)
**Sex**	
Female	17 (81.0%)
Male	4 (19.0%)
**If female, childbearing potential**	
No	9 (52.9%)
Yes	8 (47.1%)
**Ethnicity**	
Hispanic or Latino	2 (9.5%)
Not Hispanic or Latino	19 (90.5%)
**Race**	
Asian	1 (4.8%)
Black	1 (4.8%)
White	18 (85.7%)
Asian+white	1 (4.8%)
**Level of education**	
>12 years	17 (81.0%)
≤ 12 years	4 (19.0%)
**Height (cm)**	164.0 (160.0, 171.0)
**Weight (kg)**	76.0 (70.0, 86.0)

#### Compliance to treatment

The criterion for compliance was the following: achieving a minimum of 90% device usage for at least 3 hours per day over multiple sessions at a setting of 3. Nine patients (42.9%) were found to be compliant with the study device according to this criterion. Additionally, 2 patients (9.5%) achieved 80–90% of the target usage. A total of 7 patients (33.3%) had a compliance between 0 and 80% and 3 patients (14.3%) had a compliance of zero as they never used the POC for a minimum of 3 hours daily.

#### Performance on the MoCA and other neuropsychological tests

Overall, there was no relevant improvement of participants’ MoCA total score after a 2-week period under POC vs Control treatments (Table 2). The mean treatment difference was -0.090 [-1.031, 0.850]. The scores were consistently better in Period 2 regardless of the treatment sequence, which suggested a learning effect. To address this issue, post-hoc analyses were performed for Period 1 only, where the mean treatment difference in this period was 1.705 [0.140, 3.271], indicating a trend for better performance under POC treatment when compared to Control. Analyses of Period 1 MoCA subscores suggested better performances with POC for the Visuospatial/executive (0.618 [-0.106, 1.342]) and Attention subscores (0.975 [0.207, 1.743]).

**Table 2:** MoCA results.

MoCA performance according to treatment	Control			POC		
	n	Mean ± SD	Median (Q1, Q3)	n	Mean ± SD	Median (Q1, Q3)
**Total score (0–30)**						
Baseline (n = 21)						
Mean ± SD	25.38 ± 3.04					
Median (Q1, Q3)	26.00 (24.00, 28.00)					
Period1	11	24.82 ± 2.64	24.00 (23.00, 27.00)	10	27.10 ± 1.60	27.50 (26.00, 28.00)
Period2	8	28.50 ± 1.41	29.00 (27.50, 29.50)	10	25.80 ± 2.78	26.50 (25.00, 27.00)
Period1 and 2: combined	19	26.37 ± 2.85	27.00 (24.00, 29.00)	20	26.45 ± 2.31	27.00 (26.00, 28.00)
POC-Control (95%CI) [Period1]	2.282 (0.264, 4.300)					
POC-Control (95%CI) [Period2]	-2.700 (-4.999, -0.401)					
Treatment difference (n = 18)ⴕ						
Mean ± SD	0.11 ± 2.22					
Median (Q1, Q3)	0.00 (-1.00, 2.00)					
Adjusted mean (95% CI) *	-0.090 (-1.031, 0.850)					

ⴕTreatment difference: POC–Control.

*Obtained from a repeated analysis of variance (ANOVA) model with treatment group, sequence, and period as fixed effects.

Analysis performed on ITT population.

For the other neuropsychological tests, the analyses indicated that there were no notable differences in performances under POC vs Control treatments, to the exception of a trend for an advantage for POC over Control observed for the Digit Span backward test (difference 0.822 [-0.067, 1.711]; Table 3). Similar post-hoc analyses conducted on Period 1 were done for the Digit Span as it was thought to be somehow related to MoCA. This trend was also observed in the post-hoc analyses done using data from Period 1 only where the mean treatment difference in Digit Span backward test was 1.247 [-0.668, 3.161].

**Table 3:** Results of other neuropsychological tests.

Other neuropsychological test performance	Control			POC		
	n	Mean ± SD	Median (Q1, Q3)	n	Mean ± SD	Median (Q1, Q3)
**Hopkins Verbal Learning Test: Total words recalled during the three learning trials (0–36)**						
Baseline (n = 20)						
Mean ± SD	22.85 ± 5.22					
Median (Q1, Q3)	24.00 (18.50, 25.00)					
Period1	11	22.45 ± 6.07	22.00 (20.00, 25.00)	10	26.40 ± 6.15	27.00 (21.00, 32.00)
Period2	8	29.25 ± 4.06	30.50 (25.00, 32.50)	10	23.60 ± 6.43	27.00 (17.00, 28.00)
Period1 and 2: combined	19	25.32 ± 6.23	25.00 (21.00, 32.00)	20	25.00 ± 6.29	27.00 (19.50, 30.50)
POC-Control (95%CI) [Period1]	3.945 (-1.641, 9.532)					
POC-Control (95%CI) [Period2]	-5.650 (-11.203, -0.097)					
Treatment difference(n = 18)ⴕ						
Mean ± SD	0.28 ± 4.76					
Median (Q1, Q3)	1.00 (-3.00, 4.00)					
Adjusted mean (95% CI) *	-0.201 (-2.442, 2.040)					
**Hopkins Verbal Learning Test: Total words delayed recall (0–12)**						
Baseline (n = 20)						
Mean ± SD	7.00 ± 3.18					
Median (Q1, Q3)	7.50 (5.00, 9.00)					
Period1	11	7.18 ± 4.00	8.00 (6.00, 10.00)	10	9.00 ± 2.54	9.00 (7.00, 12.00)
Period2	8	10.38 ± 1.41	10.50 (9.50, 11.50)	10	7.60 ± 4.25	9.50 (2.00, 11.00)
Period1 and 2: combined	19	8.53 ± 3.50	10.00 (7.00, 11.00)	20	8.30 ± 3.48	9.00 (6.50, 11.00)
POC-Control (95%CI) [Period1]	1.818 (-1.277, 4.913)					
POC-Control (95%CI) [Period2]	-2.775 (-6.113, 0.563)					
Treatmentdifference(n = 18)ⴕ						
Mean ± SD	0.17 ± 2.20					
Median (Q1, Q3)	0.50 (0.00, 1.00)					
Adjusted mean (95% CI) *	-0.027 (-1.098, 1.044)					
**Phonological Fluency (Letter P) ‐ Total number of words (60seconds) Phonological**						
Baseline (n = 20)						
Mean ± SD	12.40 ± 3.25					
Median (Q1, Q3)	13.00 (11.00, 14.00)					
Period1	11	12.91 ± 5.24	12.00 (10.00, 16.00)	10	12.80 ± 3.01	12.50 (11.00, 16.00)
Period2	8	12.25 ± 3.88	11.50 (9.50, 13.00)	10	12.20 ± 6.58	12.50 (9.00, 16.00)
Period1 and 2: combined	19	12.63 ± 4.61	12.00 (10.00, 16.00)	20	12.50 ± 4.99	12.50 (9.50, 16.00)
POC-Control (95%CI) [Period1]	-0.109 (-4.070, 3.852)					
POC-Control (95%CI) [Period2]	-0.050 (-5.644, 5.544)					
Treatmentdifference(n = 18)ⴕ						
Mean ± SD	0.39 ± 3.97					
Median (Q1, Q3)	0.00 (-1.00, 3.00)					
Adjusted mean (95% CI) *	0.244 (-1.793, 2.281)					
**Semantic Fluency (Animals) ‐ Total number of words (60seconds) Semantic**						
Baseline (n = 20)						
Mean ± SD	17.45 ± 4.17					
Median (Q1, Q3)	16.50 (14.00, 21.00)					
Period1	11	19.45 ± 6.15	20.00 (15.00, 24.00)	10	20.30 ± 4.69	21.00 (15.00, 25.00)
Period2	8	20.38 ± 3.20	19.50 (17.50, 23.50)	10	19.10 ± 5.78	20.00 (14.00, 25.00)
Period1 and 2: combined	19	19.84 ± 5.03	20.00 (16.00, 24.00)	20	19.70 ± 5.16	20.50 (15.00, 25.00)
POC-Control (95%CI) [Period1]	0.845 (-4.193, 5.884)					
POC-Control (95%CI) [Period2]	-1.275 (-6.129, 3.579)					
Treatmentdifference(n = 18)ⴕ						
Mean ± SD	0.44 ± 2.38					
Median (Q1, Q3)	1.00 (-2.00, 2.00)					
Adjusted mean (95% CI) *	0.358 (-0.873, 1.589)					
**Digit Span Forward Total Raw Score (0–16)**						
Baseline (n = 20)						
Mean ± SD	8.65 ± 1.84					
Median (Q1, Q3)	9.00 (7.50, 10.00)					
Period1	11	8.64 ± 2.25	9.00 (7.00, 11.00)	10	9.70 ± 2.11	10.00 (8.00, 12.00)
Period2	8	9.00 ± 2.27	9.50 (7.50, 10.50)	10	8.90 ± 2.51	9.00 (8.00, 10.00)
Period1 and 2: combined	19	8.79 ± 2.20	9.00 (7.00, 11.00)	20	9.30 ± 2.30	9.00 (8.00, 11.00)
POC-Control (95%CI) [Period1]	1.064 (-0.934, 3.061)					
POC-Control (95%CI) [Period2]	-0.100 (-2.523, 2.323)					
Treatmentdifference(n = 18)ⴕ						
Mean ± SD	0.44 ± 1.15					
Median (Q1, Q3)	0.50 (0.00, 1.00)					
Adjusted mean (95% CI) *	0.443 (-0.147, 1.034)					
**Digit Span Backward Total Raw Score (0–16)**						
Baseline (n = 20)						
Mean ± SD	8.15 ± 1.93					
Median (Q1, Q3)	8.00 (6.00, 10.00)					
Period1	11	7.55 ± 2.30	8.00 (5.00, 9.00)	10	9.70 ± 2.36	8.50 (8.00, 12.00)
Period2	8	8.63 ± 1.92	9.00 (7.50, 10.00)	10	8.40 ± 2.55	7.50 (7.00, 9.00)
Period1 and 2: combined	19	8.00 ± 2.16	8.00 (7.00, 9.00)	20	9.05 ± 2.48	8.00 (7.00, 11.00)
POC-Control (95%CI) [Period1]	2.155 (0.027, 4.282)					
POC-Control (95%CI) [Period2]	-0.225 (-2.533, 2.083)					
Treatmentdifference(n = 18)ⴕ						
Mean ± SD	0.78 ± 1.73					
Median (Q1, Q3)	0.50 (0.00, 2.00)					
Adjusted mean (95% CI) *	0.822 (-0.067, 1.711)					

ⴕTreatment difference: POC–Control.

*Obtained from a repeated analysis of variance (ANOVA) model with treatment group, sequence, and period as fixed effects.

Analysis performed on ITT population.

#### Psychological health and functional status

The analyses showed that under POC, participants tended to report fewer depressive symptoms than under Control (difference -1.335 [-3.166, 0.495], Table 4). Post-hoc analyses performed on specific PHQ-9 items, namely Q1 (Little interest/pleasure) and Q7 (Trouble concentrating), also showed trends for better scores with POC, with differences of -0.374 [-0.696, -0.053] and -0.540 [-0.902, -0.179] respectively. Here again, post-hoc analyses were conducted on Period 1 for the PHQ-9. In these analyses, the mean treatment difference notably tended to be lower (better) under POC compared to Control (difference -2.246 [-4.607, 0.116]). Additionally, for the specific PHQ-9 items Q1 and Q7, participants under POC during Period 1 had lower scores (difference -1.161 [-1.820, -0.500] and -0.868 [-1.535, -0.201]; respectively. For the post-COVID functional scale (PCFS), the analyses showed a favorable trend in the ordinal scale of this questionnaire. The odds of POC having a more favorable response were 1.5 times the odds for Control (odds ratio for treatment effect: 1.501 [0.339–6.642]). Finally, the analyses showed that there were no meaningful improvements for self-reported anxiety symptoms on the GAD-7 under POC compared to Control treatment -0.168 [-2.501, 2.165].

**Table 4:** Results for psychological health and functional status tests.

Anxiety, mood, and subjective cognitive impairment	Control			POC		
	n	Mean ± SD	Median (Q1, Q3)	n	Mean ± SD	Median (Q1, Q3)
**General Anxiety Disorder-7 (GAD-7) Total Score (0–21)**						
Baseline (n = 20)						
Mean ± SD	9.45 ± 6.24					
Median (Q1, Q3)	8.00 (4.00, 16.00)					
Period1	11	11.18 ± 4.21	11.00 (9.00, 15.00)	10	7.20 ± 6.07	6.00 (2.00, 10.00)
Period2	8	5.38 ± 4.24	4.00 (2.00, 8.00)	10	8.50 ± 3.63	7.50 (5.00, 11.00)
Period1 and 2: combined	19	8.74 ± 5.05	9.00 (4.00, 13.00)	20	7.85 ± 4.91	7.50 (3.50, 10.50)
POC-Control (95%CI) [Period1]	-3.982 (-8.716, 0.753)					
POC-Control (95%CI) [Period2]	3.125 (-0.805, 7.055)					
Treatment difference (n = 18) ⴕ						
Mean ± SD	-0.22 ± 5.52					
Median (Q1, Q3)	-0.50 (-3.00, 4.00)					
Adjusted mean (95% CI) *	-0.168 (-2.501, 2.165)					
**Patient Health Questionnaire-9 (PHQ9) Total Score (0–27)**						
Baseline (n = 20)						
Mean ± SD	14.70 ± 4.32					
Median (Q1, Q3)	14.50 (11.50, 18.00)					
Period1	11	12.91 ± 3.94	13.00 (10.00, 16.00)	10	11.20 ± 5.71	12.50 (6.00, 14.00)
Period2	8	12.25 ± 4.10	12.50 (8.50, 15.00)	10	10.50 ± 4.25	11.50 (6.00, 13.00)
Period1 and 2: combined	19	12.63 ± 3.90	13.00 (9.00, 16.00)	20	10.85 ± 4.91	12.00 (6.00, 13.00)
POC-Control (95%CI) [Period1]	-1.709 (-6.152, 2.734)					
POC-Control (95%CI) [Period2]	-1.750 (-5.957, 2.457)					
Treatment difference (n = 18) ⴕ						
Mean ± SD	-1.28 ± 3.74					
Median (Q1, Q3)	-1.00 (-3.00, 1.00)					
Adjusted mean (95% CI) *	-1.335 (-3.166, 0.495)					

ⴕTreatment difference: POC–Control

*Obtained from a repeated analysis of variance (ANOVA) model with treatment group, sequence, and period as fixed effects.

Analysis performed on ITT population.

#### Cerebral oxygen saturation on NIRS

**Fig 3:**
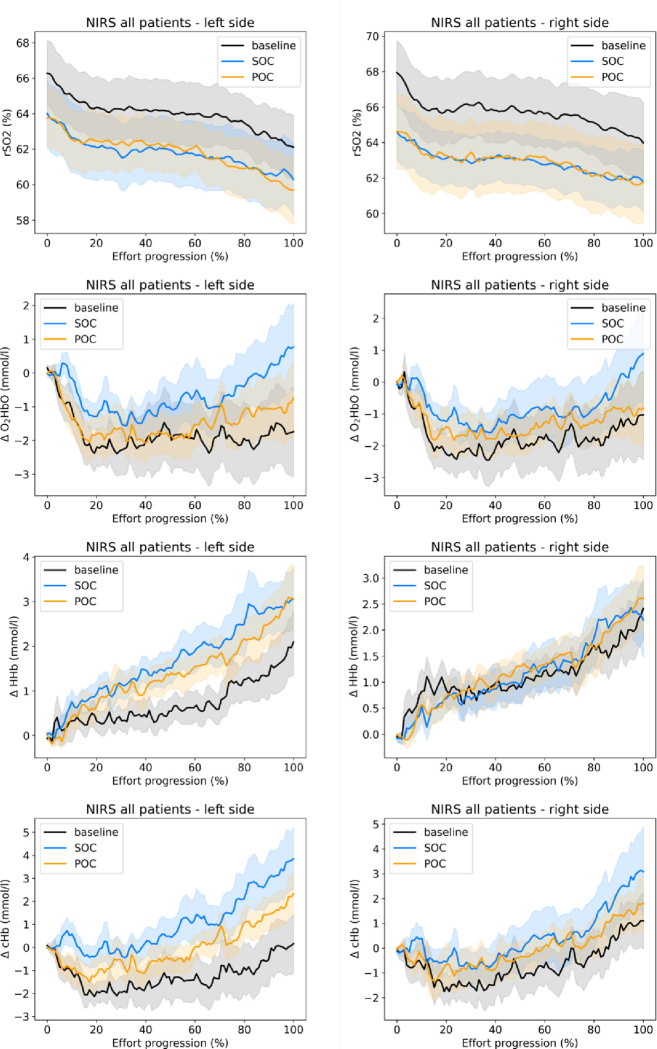
NIRS times series of rSO2, ΔO_2_Hb, ΔHHb and ΔcHb during treadmill exercise test with normalized durations, for baseline, control and POC periods. Solid lines are averages over all patients while areas indicate standard errors.

The analyses indicated that there were no noteworthy improvements in regional oxygen saturations (rSO2) under POC compared to Control treatment, both at rest and during peak effort. The mean treatment differences (POC-Control) for rSO2, with 95% confidence interval at rest were -0.503% [-2.929, 1.923] (left side) and 0.828% [-1.728, 3.384] (right side). At the end of effort, mean treatment differences (POC-Control) were -0.475% [-3.234, 2.283] (left side) and 1.043% [-1.855, 3.941] (right side). These non-significant results are mostly explained by a high inter-individual variability, as illustrated in S1 Fig. In an exploratory manner, time-series for rSO2, ΔO_2_Hb, ΔHHb and ΔcHb were averaged for all patients with their standard error, as depicted in Fig 3. Despite baseline measurements showing an overall higher saturation (tendency), all time-series showed the same dynamic pattern across treatment periods. There was an overall desaturation (rSO2 decrease) during the effort. ΔO2Hb showed a transient decrease up to 20% effort duration followed by a slow increase. Time-series of ΔHHb were increasing throughout the effort duration with an asymmetry between left and right sides. ΔHHb showed a pattern similar to ΔO2Hb (decrease followed by slow increase), but with more asymmetry between left and right sides. Finally, we report NIRS time-series for three typical cases in S2 Fig, showing different trends in response to exercise: hypoxic profile, increased arterial perfusion and arterial hypoperfusion.

#### Peripheral oxygen saturation on fingertip pulse oximetry

The analyses indicated that there were no notable improvements in peripheral oxygen saturation under POC compared to Control treatment, both at rest and peak effort. The mean treatment differences at rest and at peak effort were -0.054% [-0.787, 0.680] and 0.519% [-1.675, 2.714], respectively. Furthermore, the analysis of per-protocol (PP) patients did not yield any divergent findings. For this population, the mean treatment differences at rest and at peak effort were -0.395% [-1.921, 1.131] and -1.358% [-5.913, 3.197]; respectively.

#### Participants’ satisfaction

Participant satisfaction was assessed using a visual analog scale after the POC 2-week period. In general, patients reported positive acceptance of the POC device regarding its weight, size, and user-friendliness: 14/20 patients (70%) agreed or strongly agreed on accepting its weight and size, 19/20 patients (95%) agreed or strongly agreed that the device was user friendly and did not require mental effort to use. Also, most of patients (18/20; 90%) agreed or strongly agreed that they did not encounter any difficulty with the oxygen delivery using POC.

## Discussion

Long-COVID is a complex condition where various symptoms can persist following an acute infection with SARS-CoV-2. Cognitive impairment is among the key symptoms that are observed in individuals with long-COVID, yet, to date, no treatment has been validated. In a recent pilot retrospective study in 10 participants with long-COVID, 12 days of hyperbaric oxygen therapy (10 sessions) showed a significant reduction of perceived fatigue (Chalder scale) and improved global cognition, executive function, attention, information processing and verbal function (large effect size) [6].

We conducted an exploratory pilot crossover study designed to describe the effects of supplemental oxygen (portable oxygen concentrator, POC, Inogen) on cognitive performance, as well as on peripheral and cerebral oxygen saturation. Treatment effects were also examined for psychological health and post-COVID functional status. Twenty-one participants with long-COVID were randomized 1:1 to: 1) POC for 2 weeks followed by standard of care (Control) for 2 weeks or 2) Control for 2 weeks then POC for 2 weeks, with a 1-week washout.

First, regarding the effects of oxygen supplementation on cognitive performances, post-hoc analyses were performed for the MoCA by comparing both groups in Period 1, because participants showed learning effects on the MoCA as all participants improved their performances during Period 2, regardless of treatment sequence. These analyses suggested that participants obtained better performances under POC than Control for the total MoCA score, as well as for the visuospatial/executive and attention subscores. For the other neuropsychological tests, a possible advantage of POC over Control was only found for the Digit Span backwards test. Overall, the treatment with POC seemed to improve performance on cognitive tests that measure global cognition, attention, working memory and executive functions. Interestingly, these cognitive functions have frequently been reported as being impaired in individuals with long-COVID [3].

For psychological health, under POC, participants tended to reported fewer depressive symptoms than under Control. Post-hoc analyses of specific questions of the PHQ-9 that were hypothesized to be more sensitive to long-COVID (*“little interest/pleasure”* and *“trouble concentrating”*) tended to improve following POC treatment. Finally, POC had higher odds for lower (better) grades on the COVID functional scale when compared to Control. Altogether, POC seemed to improve psychological health and functional status. Here again, POC appeared to have an advantage over Control on symptoms frequently reported in long-COVID, mental health issues and more specifically, depressive symptoms (World Health Organization, 2022).

Finally, there were no meaningful improvements under POC compared to Control for cerebral or peripheral oxygen saturation either at rest or peak exercise. Independently of the treatment effect, we observed an overall desaturation during the exercise test. The underlying kinetics of hemoglobin changes mainly reflected an increase in total hemoglobin (cHb) suggesting an increased amount of cerebral blood volume as well as a more pronounced increase in deoxyhemoglin (HHb) compared to the decrease in oxyhemoglobin. This pattern has been observed in situations of cerebral venous congestion as a result of reduced cerebral venous drainage such as superior vena cava stenosis or acute right ventricular diastolic dysfunction which could have been triggered by exercise as in patients with chronic lung disease [19]. We also observed asymmetrical patterns for cHB and HHb time-series between left and right sides. Such differences are rarely reported [20, 21] and could indicate underlying neurological disorders as reported in pre-term infants [22].

Although the results from this study are promising, some limitations need to be considered. First, the study was exploratory, thus the sample size was small and of convenience. This is however the essential first step in order to determine future adequately sized trials. Future studies with a larger sample size are needed to confirm these findings. Also, because of the learning effects that were observed on the MoCA, post-hoc analyses were performed. Moreover, performances on the MoCA at baseline were mostly in the normal range for participants in both sequences (> 26/30), which could have limited the range for possible improvement. Future studies with cognitive tests that are less sensitive to learning effects, that are more challenging or that are administered over a longer period (longer than 2 weeks) are needed to reproduce these results and better understand the phenomenon. Brain oximetry signals were obtained from two small areas from the frontal cortex. Alterations in those regions may not correlate with those from which neuropsychological anomalies do occur. Here again, participants showed values of cerebral oxygen saturation in the normal range SpO2 (≥ 60%), which could have limited the opportunity for improvement. Neuro-vascular coupling assessed by oximetry and a cognitive task with a larger interrogated brain area might have been more sensitive to detect the benefit of a therapeutic intervention [23]. Finally, the present study had a short follow-up of 2 weeks, thus the effects of longer-term oxygen supplementation on cognition and psychological health are unknown. Future studies are needed to determine the long-term effects of oxygen supplementation in long-COVID.

Overall, our study revealed possible benefits of POC on cognitive performance in domains that are affected by long-COVID, as well as for depressive symptoms and functional status. Studies are needed to validate interventions for the management of long-COVID.

## Supporting information

S1 Fig Boxplots of NIRS changes from rest to end of effort of rSO2, ΔO_2_Hb, ΔHHb and ΔcHb for all patients and treatments. Solid lines represent individual trajectories.
(TIF)

S2 Fig Typical cases of NIRS times series of rSO2, ΔO_2_Hb, ΔHHb and ΔcHb during treadmill exercise test for 3 patients. Patient #12 (Control) showed a normal increase of cerebral arterial perfusion during exercise: slight rSO2 increase, marked increase of O2HHb and cHB with stable HHb. Patient #12 showed a hypoxic profile: rSO2 decrease, O2HHb decrease and HHb increase with stable cHb. Patient #05 (baseline) showed an arterial hypoperfusion profile: decrease of rSO2, O2HHb and cHb, with an increase in HHb.
(TIF)

S1 Dataset (XLSX)

## References

[1] Davis HE, McCorkell L, Vogel JM, Topol EJ. Long COVID: major findings, mechanisms and recommendations. Nat Rev Microbiol. 2023;21(3):133–146. doi: 10.1038/s41579-022-00846-2 36639608 PMC9839201

[2] World Health Organization. Post COVID-19 condition (Long COVID) [Internet]. 2022. Available from: https://www.who.int/europe/news-room/fact-sheets/item/post-covid-19-condition

[3] Quan M, Wang X, Gong M, Wang Q, Li Y, Jia J. Post-COVID cognitive dysfunction: current status and research recommendations for high risk population. Lancet Reg Health West Pac. 2023;38:100836. doi: 10.1016/j.lanwpc.2023.100836 37457901 PMC10344681

[4] Østergaard L. SARS CoV-2 related microvascular damage and symptoms during and after COVID-19: Consequences of capillary transit-time changes, tissue hypoxia and inflammation. Physiol Rep. 2021;9(3):e14726. doi: 10.14814/phy2.14726 33523608 PMC7849453

[5] Delgado-Alonso C, Valles-Salgado M, Delgado-Álvarez A, Yus M, Gómez-Ruiz N, Jorquera M, et al. Cognitive dysfunction associated with COVID-19: A comprehensive neuropsychological study. J Psychiatr Res. 2022;150:40–46. doi: 10.1016/j.jpsychires.2022.03.033 35349797 PMC8943429

[6] Robbins T, Gonevski M, Clark C, Baitule S, Sharma K, Magar A, et al. Hyperbaric oxygen therapy for the treatment of long COVID: early evaluation of a highly promising intervention. Clin Med Lond Engl. 2021;21(6):e629–e632. doi: 10.7861/clinmed.2021-0462 34862223 PMC8806311

[7] Jessen F, Amariglio RE, Buckley RF, van der Flier WM, Han Y, Molinuevo JL, et al. The characterisation of subjective cognitive decline. Lancet Neurol. 2020;19(3):271–278. doi: 10.1016/S1474-4422(19)30368-0 31958406 PMC7062546

[8] Gagnon C, Olmand M, Dupuy EG, Besnier F, Vincent T, Grégoire CA, et al. Videoconference version of the Montreal Cognitive Assessment: normative data for Quebec-French people aged 50 years and older. Aging Clin Exp Res. 2022;34(7):1627–1633. 35178685 10.1007/s40520-022-02092-1 PMC8853900

[9] Nasreddine ZS, Phillips NA, Bédirian V, Charbonneau S, Whitehead V, Collin I, et al. The Montreal Cognitive Assessment, MoCA: a brief screening tool for mild cognitive impairment. J Am Geriatr Soc. 2005;53(4):695–699. doi: 10.1111/j.1532-5415.2005.53221.x 15817019

[10] Benedict R, Schretlen D, Groninger L, Brandt J. Hopkins Verbal Learning Test- Revised. PARinc; 1991. 10.1076/clin.13.3.348.1749 10726605

[11] Wechsler D. WAIS-IV Wechsler Adult Intelligence Scale. 2008.

[12] Spitzer RL, Kroenke K, Williams JBW, Löwe B. A brief measure for assessing generalized anxiety disorder: the GAD-7. Arch Intern Med. 2006;166(10):1092–1097. doi: 10.1001/archinte.166.10.1092 16717171

[13] Kroenke K, Spitzer RL, Williams JB. The PHQ-9: validity of a brief depression severity measure. J Gen Intern Med. 2001;16(9):606–613. doi: 10.1046/j.1525-1497.2001.016009606.x 11556941 PMC1495268

[14] Machado FVC, Meys R, Delbressine JM, Vaes AW, Goërtz YMJ, van Herck M, et al. Construct validity of the Post-COVID-19 Functional Status Scale in adult subjects with COVID-19. Health Qual Life Outcomes. 2021;19(1):40. doi: 10.1186/s12955-021-01691-2 33536042 PMC7856622

[15] World Health Organization. The WHO Global Clinical Platform for COVID-19. 2023.

[16] Trachsel LD, Nigam A, Fortier A, Lalongé J, Juneau M, Gayda M. Moderate-intensity continuous exercise is superior to high-intensity interval training in the proportion of VO2peak responders after ACS. Rev Espanola Cardiol Engl Ed. 2020;73(9):725–733. doi: 10.1016/j.rec.2019.09.013 31837947

[17] Calderone A, Jarry S, Couture EJ, Brassard P, Beaubien-Souligny W, Momeni M, et al. Early Detection and Correction of Cerebral Desaturation With Noninvasive Oxy-Hemoglobin, Deoxy-Hemoglobin, and Total Hemoglobin in Cardiac Surgery: A Case Series. Anesth Analg. 2022;135(6):1304–1314. doi: 10.1213/ANE.0000000000006155 36097147

[18] Savitzky A, Golay M. Smoothing and Differentiation of Data by Simplified Least Squares Procedures. Anal Chem. 1964;36(8):1627–1639.

[19] Fenster BE, Holm KE, Weinberger HD, Moreau KL, Meschede K, Crapo JD, et al. Right ventricular diastolic function and exercise capacity in COPD. Respir Med. 2015;109(10):1287–1292. doi: 10.1016/j.rmed.2015.09.003 26371994 PMC4745988

[20] Eyeington CT, Ancona P, Osawa EA, Cutuli SL, Eastwood GM, Bellomo R. Modern technology-derived normative values for cerebral tissue oxygen saturation in adults. Anaesth Intensive Care. 2019;47(1):69–75. doi: 10.1177/0310057X18811962 30864480

[21] Misra M, Stark J, Dujovny M, Widman R, Ausman JI. Transcranial cerebral oximetry in random normal subjects. Neurol Res. 1998;20(2):137–141. doi: 10.1080/01616412.1998.11740496 9522349

[22] Lemmers PMA, van Bel F. Left-to-right differences of regional cerebral oxygen saturation and oxygen extraction in preterm infants during the first days of life. Pediatr Res. 2009;65(2):226–230. doi: 10.1203/PDR.0b013e318191fb5d 18948838

[23] Talamonti D, Gagnon C, Vincent T, Nigam A, Lesage F, Bherer L, et al. Exploring cognitive and brain oxygenation changes over a 1-year period in physically active individuals with mild cognitive impairment: a longitudinal fNIRS pilot study. BMC Geriatr. 2022;22(1):648. doi: 10.1186/s12877-022-03306-x 35941561 PMC9361664

