# Supplementary figures and images for: Oxygen supplementation and cognitive function in long-COVID
Source: PLoS One. 2024 Nov 5;19(11):e0312735. doi: 10.1371/journal.pone.0312735 (PMC11537380; doi:10.1371/journal.pone.0312735)

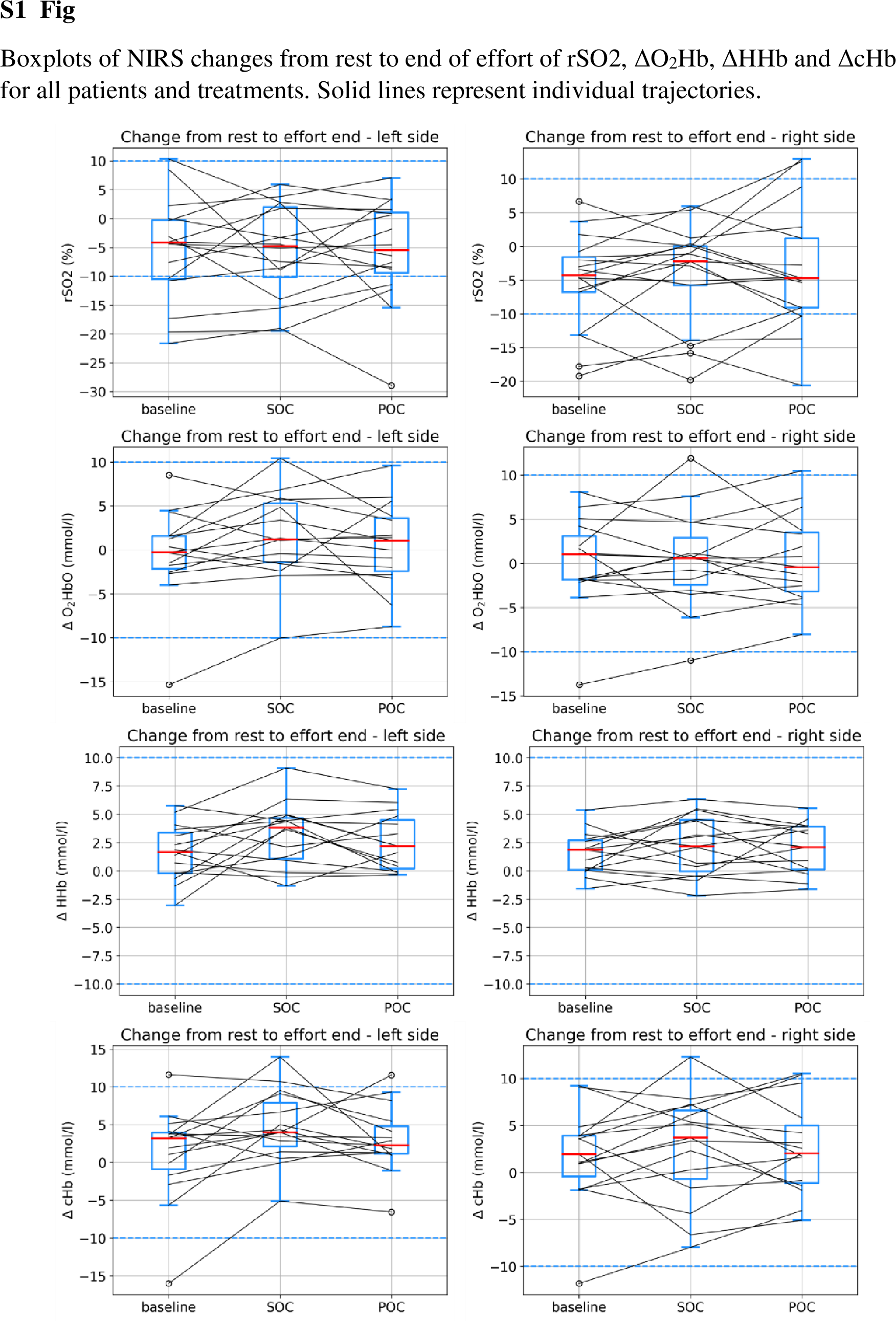

Supplement: S1 Fig — Solid lines represent individual trajectories. (TIF) [file pone.0312735.s001.tif]

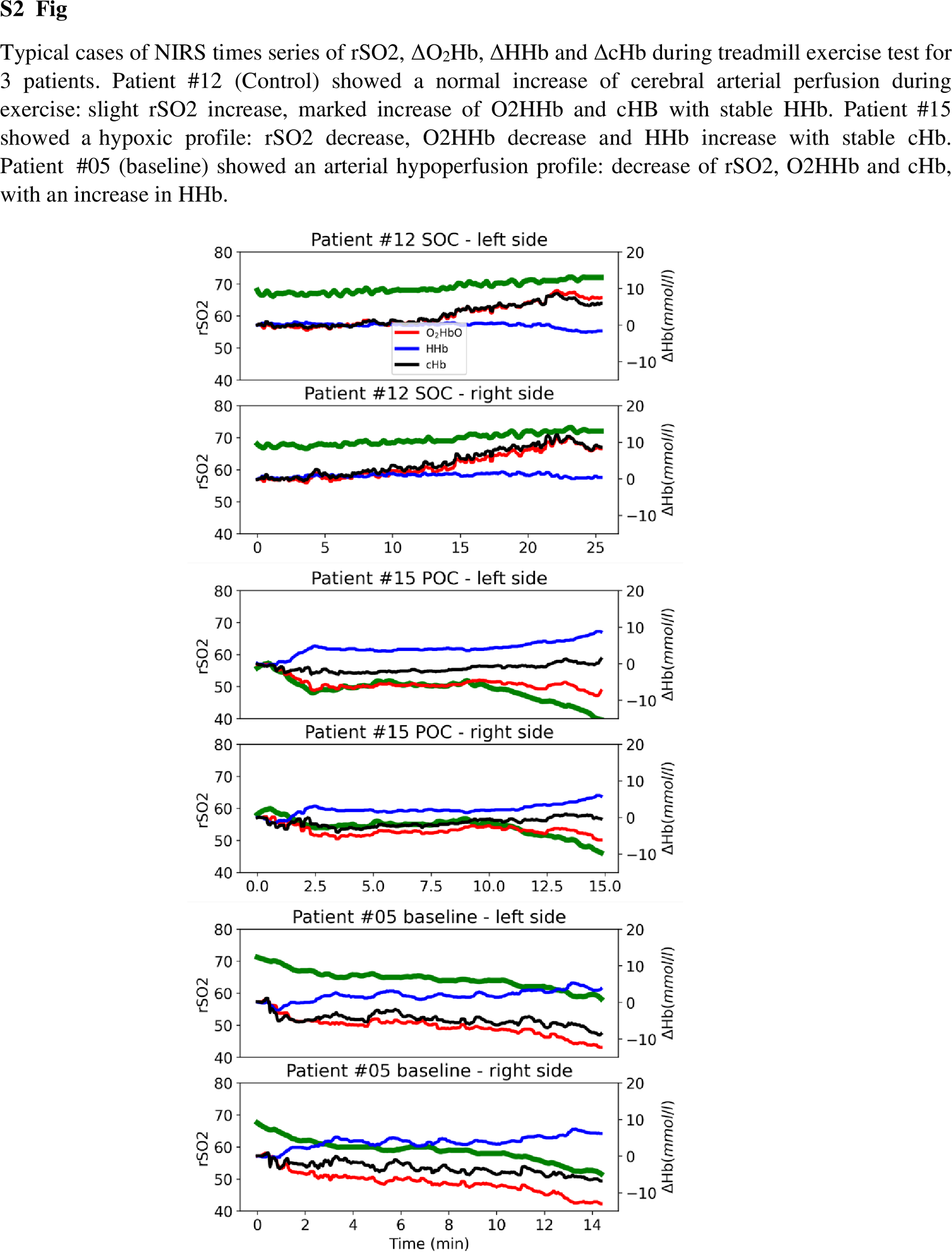

Supplement: S2 Fig — Patient #12 (Control) showed a normal increase of cerebral arterial perfusion during exercise: slight rSO2 increase, marked increase of O2HHb and cHB with stable HHb. Patient #12 showed a hypoxic profile: rSO2 decrease, O2HHb decrease and HHb increase with stable cHb. Patient #05 (baseline) showed an arterial hypoperfusion profile: decrease of rSO2, O2HHb and cHb, with an increase in HHb. (TIF) [file pone.0312735.s002.tif]
